# Clinical Evaluation of CAD/CAM Ceramic Endocrown Versus Prefabricated Zirconia Crown in the Restoration of Pulpotomized Primary Molars: A Two-Year Spilt-Mouth Randomized Controlled Trial

**DOI:** 10.1055/s-0041-1736417

**Published:** 2022-02-23

**Authors:** Nagwa Mohmmad Ali Khattab, Yasmine Mohamed Farouk El Makawi, Ahmad Abdel Hamid Elheeny

**Affiliations:** 1 Pediatric and Community Dentistry Department, Faculty of Dentistry, Ain Shams University, Cairo, Egypt; 2 Pediatric Dentistry Department, Faculty of Oral and Dental Medicine, Nahda University, Beni Suef, Egypt; 3 Pediatric and Community Dentistry Department, Faculty of Dentistry, Minia University, Minya, Egypt

**Keywords:** adhesive, pediatric dentistry, aesthetic, crowns, gingivitis

## Abstract

**Objectives** The current trial aimed to compare lithium disilicate (LS2) endocrowns' clinical performance, gingival health, and parental satisfaction to those of prefabricated zirconia crowns (ZCs) over a 24-month of follow-up.

**Materials and Methods** This study designed as a spilt-mouth randomized controlled trial. A total of 88 pulpotomized mandibular second primary molars of 44 children were assigned into two equal groups. Forty-four molars were restored with prefabricated primary ZCs (control group) and the same number were restored with LS2 endocrown (intervention group). Clinical performance and gingival status were evaluated using a modified United States Public Health Service criterion, and plaque and gingival indices. Parental satisfaction was assessed using a 5-point Likert-scale questionnaire.

**Statistical Analysis** Paired data were analyzed using McNemar's test, a statistical test used on paired nominal data, and paired *t* -tests. The significance level was set to 5% at 95% confidence interval.

**Results** Both restorations showed comparable gingival health status over the follow-ups. Marginal adaptation of the endocrowns and ZCs at the end of follow-up was 95.5 and 90.9%, respectively ( *p* = 0.68). For marginal integrity and discoloration, both restorations showed similar results at the follow-ups. The overall parental satisfaction of both groups was statistically insignificant ( *p* = 0.07). However, parents were more satisfied with the endocrown color over that of the ZC ( *p* < 0.05).

**Conclusion** Endocrowns' clinical performance and gingival health were comparable to those of ZCs. For both restorations, parental satisfaction was nearly similar except for the color that showed an advantage in favor of the endocrowns.

## Introduction

Restoration of pulpotomized primary molars is a commonly performed procedure in the pediatric dental office. Using a suitable final restoration is one of the determinants of success or failure of endodontically treated primary molars. 1 Restoration of pulpotomized primary molars serves to replace the lost tooth structure and enhance mechanical and functional properties that augment the long-term prognosis and survival of the tooth. 2 Stainless steel crowns (SSCs) are the commonly used full-coverage restoration of the primary molars. SSCs are characterized by their high durability, minimum technique sensitivity during placement, and their reasonable cost. 3 However, aesthetic restorations are often required to satisfy both patients and parents/guardians.

A variety of commercially available aesthetic crowns (both veneered SSC and zirconia crowns [ZCs]) chosen for primary molars are available, such as NuSmile (Houston, Texas, United States), Kinder Krowns (St Louis Park, Minnesota, United States), EZCrowns (Sprig Oral Health Technologies), and Cheng Crown (Exton, Pennsylvania, United States). 4 Prefabricated ZCs of zirconia-toughened alumina-type Y-TZP emerged in 2008 for anterior and posterior primary dentition. Besides their superior aesthetic properties and low thermal conductivity, their mechanical properties are comparable to metal crown properties such as high fracture resistance. 5

For primary molars, an adequate reduction is a determinant factor of ZC's passive fitting. Tooth reduction to receive ZCs is more aggressive than that required for SSCs by approximately 20 to 30%. 6 The clinical performance of NuSmile ZCs versus SSCs was evaluated in a previous study conducted on 120 primary molars of 26 children. The results showed a 100% clinical success rate of ZCs during a follow-up period of 12 months. Another two prospective studies comparing ZCs and SSCs over 24 and 36 months showed no clinical signs of failure in terms of gingival status and different parameters regarding crown integrity. 7 8 The gingival bleeding index scores of ZCs were lower than the records reported for SSCs, indicating that teeth restored with ZCs showed a better gingival status. 9 Another study compared the periodontal health and clinical success of 52 primary molars restored with ZCs in comparison with SSCs. After a 12-month follow-up, two ZCs were dislodged and one crown was fractured while all SSCs were retained. Nevertheless, the ZCs' periodontal health was better than that of SSCs. 10

With the emergence of adhesive restorations and their ability to support the remaining tooth structure, more conservative approaches have been considered to preserve as much as possible of natural tooth structures. One of these strategies for permanent teeth is the use of endocrowns which were first described in 1999 by Bindl and Mörmann. Endocrowns are monolithic adhesive ceramic restorations with a special preparation design, including a butt joint preparation at the cervical margin and pulp chamber preparation. 11 Their retention is gained macro-mechanically by friction with the pulpal walls, and micromechanically through adhesive cementation. 12 Commonly, glass-ceramic endocrowns are made of lithium disilicate (LS2) which is pressed or CAD/CAM milled. LS2 is responsible for the high mechanical performance and excellent biocompatibility of the glass-ceramic endocrown. 13 Using glass-ceramic endocrowns for restoration of endodontically treated permanent teeth has become a popular approach, and there are an abundance of reviews discussing the application of ceramic endocrowns in restoring permanent endodontically treated teeth. In contrast, there were no previous clinical trials concerned with restoring primary molars with ceramic endocrowns. Despite the aesthetic properties of ZCs and LS2 ceramic endocrowns, LS2 ceramic endocrowns' aesthetic and translucency are 30% more superior to ZCs. 14 Ceramic endocrowns give the opportunity of color matching, and shade selection that is not available in ZCs. Endocrowns need less circumferential tooth reduction than ZCs, but the occlusal surface reduction is similar for both restorations. Finally, the endocrown preparation ends supragingival that eliminates any possible discomfort or gingival trauma during tooth reduction. In contrast, the finishing line of ZC must extend for 1 to 2 mm subgingivally.

Clinical application of restoring pulpotomized primary molars with ceramic endocrowns is a novel approach. The present trial aimed to evaluate (1) the clinical performance, assessment of dental plaque, and gingival status of LS2 endocrowns as a new restorative modality in pulpotomized primary molars compared with prefabricated ZCs, and (2) the parental satisfaction toward the two restorations at the end of the 24-month follow-up. The primary outcomes of the current study are whether the LS2 endocrowns' gingival health and clinical performance differ from those of prefabricated ZCs over 24 months.

## Materials and Methods

### Ethical Considerations

The study was reviewed and approved by an institutional review board (Record number #186/2016). Informed consent was obtained from all children's parents/guardians. The trial was registered at ClinicalTrials.gov PRS (Protocol registration and Result System) with an ID of NCT04073901. The study procedures involving human participants followed the ethical standards of Helsinki Declaration in 1964 and its later amendments or comparable ethical standards.

### Sample Calculation, Design, and Setting

The study was designed as a two-tailed split-mouth randomized controlled trial. The sample size for repeated-measures of longitudinal studies was calculated using the following software “General Linear Mixed Model Power and Sample Size” (GLMMPSS) (glimmpse.samplesizeshop.org/). 15 Specification of the mean value of the repeated measures (i.e., at three follow-up occasions) and the standard deviation (SD) calculated on the base of gingival index (GI) score results of a pilot study included 13 children. 16 The chosen power was 0.8 and at 0.05 level of significance. The required sample size was 80 teeth with adding more 8 teeth (10%) to compensate unexpected withdrawal. The final number of recruited children was 44 children (88 bilateral second primary molars).

### Admission Specifications

Children's admission in the present study based on eligibility criteria addressed in a former study. 9

### Inclusion Specifications

- Apparently healthy children (i.e., class I and II relying on American Society of Anesthesiologists' classification).
- Participant's age ranging from 4 to 6 years old.
- Cooperative child.
- Presence of bilateral deep carious lesion on mandibular second primary molar requiring pulpal therapy with at least two-thirds of root length was sound on a periapical radiograph.

### Exclusion Specifications

- Presence of parafunctional habits.
- Intellectual behavior and/or severe emotional difficulties.
- Parents/legal guardians refused to take part in the trial.
- Tooth with preshedding and abnormal pathologic mobility.
- Nonrestorable tooth.
- Teeth with nonvital pulp.

### Randomization, Allocation, and Masking

**Fig. 1:**
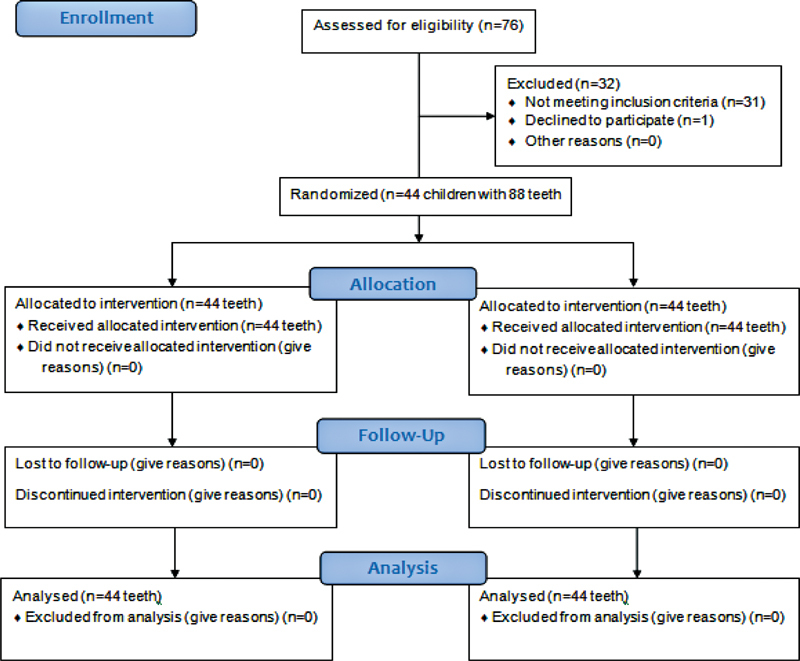
Flow chart of the trial enrollment, randomization, allocation, and analysis throughout the follow-up period according to CONSORT guidelines.

The study was conducted in accordance with the Consolidated Standards of Reporting Trials (CONSORT) statement ( Fig. 1 ). An independent coordinator was responsible for generating a randomization sequence and keeping the sequence hidden. Children were numbered in consecutive ascending order. The two restorations were randomly assigned either to the right or the left side masked to the chief operator. An opaque hermetically sealed envelope containing a printed letter including patient's ID, code, name, time, date, tooth number, and restoration type was used. The envelope was not opened by the operator until the first visit. 17 It is always decided to begin with the endocrown procedures in the first visit because it is a two-appointment procedure, while the ZC takes one-appointment only. As operator and patient blinding cannot be achieved, the treatment nature was masked for the statistician to avert detection bias. In addition, two independent experts with at least 10 years of experience in pediatric dentistry and 7 years of ZC placement were invited to assess the outcomes in two different occasions (1-week interval) to check the intra- and inter-observer reliability. According to the restoration type, the mandibular primary molars were assigned into two balanced groups. Group 1 (control group): teeth restored with prefabricated primary ZCs (NuSmile Ltd., Houston, Texas, United States). Group 2 (intervention group): the primary molars restored with laboratory-processed endocrown (IPS, e.max Press, Ivoclar Vivadent, Liechtenstein, Schaan, Germany).

### Clinical Steps

For each child, two visits were required. In the first visit, pulpotomy and endocrown preparation and impression procedures were performed for the randomly selected primary molar. After 1-week interval, pulpotomy and ZC placement of the contralateral primary molar and endocrown try-in and cementation were performed in the second visit. This was to ensure a parallel starting point for the follow-up period of both restorations.

### Intervention Group

#### Pulpotomy Procedures

The tooth was anaesthetized using articaine hydrochloric 4% with epinephrine 1:100,000 (Septocaine 1.7 mL, SEPTODONT Ltd., Canada). Then, it was isolated using a rubber dam after caries removal and a non-end cutting bur # 558 was used to complete the removal of the pulp chamber roof under copious water coolant spray. Coronal pulp tissue remnants were removed with a sharp, sterile excavator. A piece of cotton soaked with formocresol (Sultan, United States) was inserted into the pulp chamber for 5 minutes.

After removing the formocresol pellet, a thick mix of polymer reinforced zinc-oxide/eugenol (ZOE; Zinconol, Prevest DenPro, India) paste was packed into the pulp chamber to seal the orifices. For the endocrown, a layer of self-cured glass ionomer cement (GIC; Riva Self Cure, Australia) of 1 mm thickness was applied over the ZOE leaving a minimum of 3 mm of the pulp chamber to provide an adequate thickness for the endocrown core. The GIC was applied over the capping ZOE to isolate it from the successive resin-based restorations and adhesives.

#### Endocrown Tooth Preparation

Occlusal clearance was achieved by making depth cuts of 1.5 mm using a round-end tapered stone (TR-13, DIA-Burs Mani, Inc., Japan). A wheel stone (WR-13, DIA-Burs Mani, Inc., Japan) completed the occlusal reduction and prepared a cervical sidewalk or “butt joint” finish line. Axial preparation was done to eliminate proximal wall undercut. Tapered stone of 8-degree angle (TR-12 DIA-Burs Mani, Inc., Japan) flared the pulp chamber walls to a standard degree of divergence. Smoothening and rounding the internal angles of the margins began with the use of the same diamond tip and ended with a polishing of the internal angles with an abrasive rubber tip giving a polished and smoothed preparation.

#### Shade Selection, Impression, and Temporization

Based on a shade guide (Vita, Classic, Germany), the suitable ceramic shade was selected. A putty and light body additional silicon impression was taken for the prepared pulp chamber and occlusal butt joint finish line. An alginate impression was taken for the opposing arch, then a squash bite with pink wax was sent to the laboratory. An interim prosthesis with self-curing resin (Protemp 3M, United States) temporized the prepared tooth and was cemented with eugenol-free temporary cement (Ora temp, United States).

#### Laboratory Procedures

**Fig. 2:**
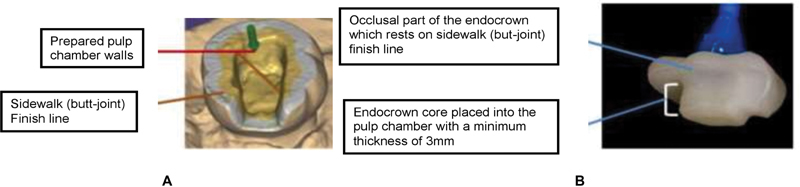
( **A** ) Exocad showing endocrown preparation of pulpotomized primary molar; ( **B** ) intaglio surface of lithium disilicate (LS2) endocrown (crown and core act as a single unit).

A 5-axis milling machine (VHF CAM 5-S1) for indirect fabrication of the wax pattern scanned the impression. All CAD wax patterns were sprued and invested in an investment material powder (IPS Press VEST, Liechtenstein) that was mixed with its special liquid under vacuum according to the recommended manufacturer's proportions, then poured around the wax patterns inside the investment ring. The investment was left to set for 1 hour before wax elimination. The wax burn-out technique was used for wax elimination with pressing of IPS e.max Press ingot (Ivoclar-Vivadent AG, Liechtenstein) in a pressable furnace (Programat EP 3010, Liechtenstein). Finally, IPS e.max Press endocrowns were consecutively finished using a low-speed fine-grained diamond and were checked for occlusion, then were glazed using IPS e.max ceram ( Fig. 2 ).

#### Try-In, Bonding, and Occlusion Re-check

At the second visit, the endocrown was placed into the tooth and occlusion was initially checked. Then the tooth was treated according to the following steps: (1) proper tooth isolation using a rubber dam after intrapapillary injection with a local anesthetic agent, (2) selective etching using 37% phosphoric acid for 20 seconds, (3) thorough rinsing and air drying, and (4) application of separate coats of self-etch adhesive (universal bond) (RelyX Unicem; 3M, St. Paul, Minnesota), then dried with oil-free air for 3 seconds and light-cured for 20 seconds. A micro brush with 4% hydrofluoric acid gel (Bisco, Inc., United States) was applied to the endocrown for 20 seconds then rinsed for 60 seconds and dried for 30 seconds with moisture-free air. Following the manufacturer's instructions, a dual-cured, self-adhesive resin cement (TOTALCEM, Itena, France) was applied to the endocrown internal surface and the prepared tooth walls. The restoration was gently set in place using digital pressure, then initially cured with LED light cure (1,200 mW/cm ^2^ ) (Elipar, 3M ESPE, Canada) for 3 seconds. Excess cement removed with a scaler and super floss and final curing was continued for another 40 seconds. Occlusion was re-checked for premature spots with an articulating paper. Any further occlusal adjustments were performed using a medium-grit egg-shaped ZR379FG-14 diamond bur. Finally, the endocrown was polished with a light-gray flame and cup-shaped ZR Flash Polishers (94020C RA.040 Komet Dental, United States). 18

### Control Group

At the second visit, anesthesia, isolation, and pulpotomy procedures were identical to the first visit. The pulp chamber was completely filled with GIC (Riva Self Cure, Australia) to the top. (1) A suitable crown size was identified using a NuSmile (Houston, Texas, United States) Try-In Crowns, (2) the tooth was prepared with 1.5 mm occlusal clearance using TR-13 and WR-13 diamond burs, (3) supragingival circumferential tooth preparation was performed by approximately 20 to 30% to guarantee reduction evenness, then preparation was extended subgingivally (1.5 mm) into a smooth feather-edged cervical margin to ensure a passive crown fit, (4) Try-In Crowns (pink crowns) were used to accurately determine passive fit without applying significant pressure during seating and for checking occlusion to avoid saliva or blood contamination of the NuSmile ZCs internal surface, and (5) finally, the ZC was cemented with self-adhesive dual cure resin cement similar to the endocrown.

### Clinical Performance Assessment

For proper examination, sufficient overhead lighting, a mouth mirror, and a sickle probe (No. 23) were used. The clinical performance in terms of marginal adaptation, marginal discoloration, and marginal integrity of the restoration were scored using a United States Public Health Service (USPHS) criterion. The USPHS scoring system was described on the basis of previous literature. 8 19 Alpha and Bravo scores were considered a success, while Charlie was a failure. Dental plaque accumulation and gingival condition were assessed using plaque index (PI) and GI. 20 To assess the gingival health status, four areas (distal-facial papilla, facial margin, mesial-facial papilla, and the entire lingual margin) around the restored tooth were examined. The involved tooth was first examined for dental plaque accumulation using the naked eye and a diagnostic dental probe. Clinical performance and oral status were assessed at follow-up periods of 6 (T1), 12 (T2), and 24 (T3) months. At the end of the follow-up (T3), parent's satisfaction analysis toward the color, shape, and size of both restorations was adopted from previous literature. 21 22 In absence of the chief investigator, an independent pediatric dentist with an experience of at least 5 years was responsible for reporting the parent's answers to the anonymous questionnaire aimed to directly evaluate their satisfaction toward their children's restorations. Parents' responses were rated on a 5-point Likert-type scale ranging from score “1” which indicated that they were strongly dissatisfied, up to score “5” which equaled “strongly satisfied.” The details of different study outcomes are illustrated in Appendix A1 ( Appendix Tables A1–A4 , available in the online version only).

### Statistical Data Processing

Data processing was implemented using Statistical Package for the Social Sciences (SPSS) version 20. GI and PI scores were first tested for normality using Kolmogorov–Smirnov and Shapiro–Wilk tests. Paired-sample *t-* test was used to compare the mean values of normally distributed GI and PI scores. Success or failure of both restorations in terms of gingival and plaque status was interpreted through dichotomizing the GI and PI scores as follows: no/mild plaque or gingivitis was categorized “success,” while moderate/sever plaque accumulation or gingivitis were categorized “failure.” Additionally, the USPHS scores (Alpha, Bravo, and Charlie) were expressed as frequencies, then dichotomized into binary outcome USPHS criteria (Alpha/Bravo was “success,” while Charlie was “failure”). Pair matched binary outcomes were tested using McNemar's test. Regarding parental satisfaction, responses were classified into five categories (very unsatisfied scored “1,” unsatisfied “2,” neutral “3,” satisfied “4,” and very satisfied “5”). Paired data scores of parents' responses for the two restorations were tested for significance using Friedman test. The level of significance for all statistical tests (α level of significance) was set to 5% at 95% confidence interval (CI).

## Results

Children's recruitment was started on January 2017 and completed on October 2020. Out of 92 children examined for eligibility, 44 were selected for participation in the trial (21 girls [47.7%] and 23 boys [52.3%]). Their average age ± SD was 5.17 ± 0.68 years. The intra-and inter-observer reliability of both assessors was high (96 and 92%, respectively).

Marginal discoloration and integrity were identical for both restorations throughout the follow-up period. At 6 months, all restorations in the control and intervention groups were ranked as “Alpha.” Regarding marginal adaptation, 100% of restorations showed marginal continuity of ZCs and endocrowns at 6 months when assessed by an explorer. At 12 and 24 months, marginal adaption of both restorations displayed no evidence of a crevice along the restoration margin. Generally, the clinical success rate after 24 months of follow-up period of the two restorations was comparable ( Table 1 ).

**Table 1:** Frequency of clinical performance of the endocrown and zirconia crown groups at different follow-up periods

Variables	At 6 mo		*p* a	At 12 mo		*p* a	At 24 mo		*p* a
	Endocrown	ZC		Endocrown	ZC		Endocrown	ZC	
	*N* (%)	*N* (%)		*N* (%)	*N* (%)		*N* (%)	*N* (%)	
Marginal adaptation Alpha Bravo Charlie	44 (100) 0 (0) 0 (0)	44 (100) 0 (0) 0 (0)	1	43 (97.7) 1 (2.3) 0 (0)	42 (95.5) 2 (4.5) 0 (0)	0.56	42 (95.5) 2 (2.5) 0 (0)	40 (90.9) 4 (9.1) 0 (0)	0.68
Marginal discoloration Alpha Bravo Charlie	44 (100) 0 (0) 0 (0)	44 (100) 0 (0) 0 (0)	1	44 (100) 0 (0) 0 (0)	44 (100) 0 (0) 0 (0)	1	44(100) 0(0) 0(0)	44 (100) 0 (0) 0 (0)	1
Marginal integrity Alpha Bravo Charlie	44 (100) 0 (0) 0 (0)	44 (100) 0 (0) 0 (0)	1	44 (100) 0 (0) 0 (0)	44 (100) 0 (0) 0 (0)	1	44 (100) 0 (0) 0 (0)	44 (100) 0 (0) 0 (0)	1

Abbreviation: ZC, zirconia crown.

a McNemar's test, level of significance (α) was set to 5% ( *p* < 0.05 for two-tailed test).

Throughout the follow-up period, gingival and plaque health showed no statistically significant difference between average PI and GI scores of ZC and endocrown groups ( *p* > 0.05) ( Table 2 ).

**Table 2:** Frequency of gingival health in terms of PI and GI of the endocrown and zirconia crown groups at different follow-up periods

Variables	At 6 mo		*p* a	At 12 mo		*p* a	At 24 mo		*p* a
	Endocrown	ZC		Endocrown	ZC		Endocrown	ZC	
	*N* (%)	*N* (%)		*N* (%)	*N* (%)		*N* (%)	*N* (%)	
Plaque index Excellent Good Fair Poor	38 (86.4) 6 (13.6) 0 (0) 0 (0)	41 (93.3) 3 (6.8) 0 (0) 0 (0)	0.48	39 (88.6) 4 (9.1) 1 (2.3) 0 (0)	41 (93.3) 3 (6.8) 0 (0) 0 (0)	0.71	39 (88.6) 5 (11.4) 0 (0) 0 (0)	39 (88.6) 5 (11.4) 0 (0) 0 (0)	1
Gingival index Normal Mild gingivitis Moderate gingivitis Sever gingivitis	43 (97.7) 1 (2.3) 0 (0) 0 (0)	43(97.7) 1(2.3) 0(0) 0 (0)	1	41 (93.2) 3 (6.8) 0 (0) 0 (0)	42 (95.5) 2 (4.5) 0 (0) 0 (0)	1	41 (93.2) 3 (6.8) 0 (0) 0 (0)	42 (95.5) 2 (4.5) 0 (0) 0 (0)	1

Abbreviations: GI, gingival index; PI, plaque index; ZC, zirconia crown.

a McNemar's test, level of significance (α) was set to 5% ( *p* < 0.05 for two-tailed test).

**Fig. 3:**
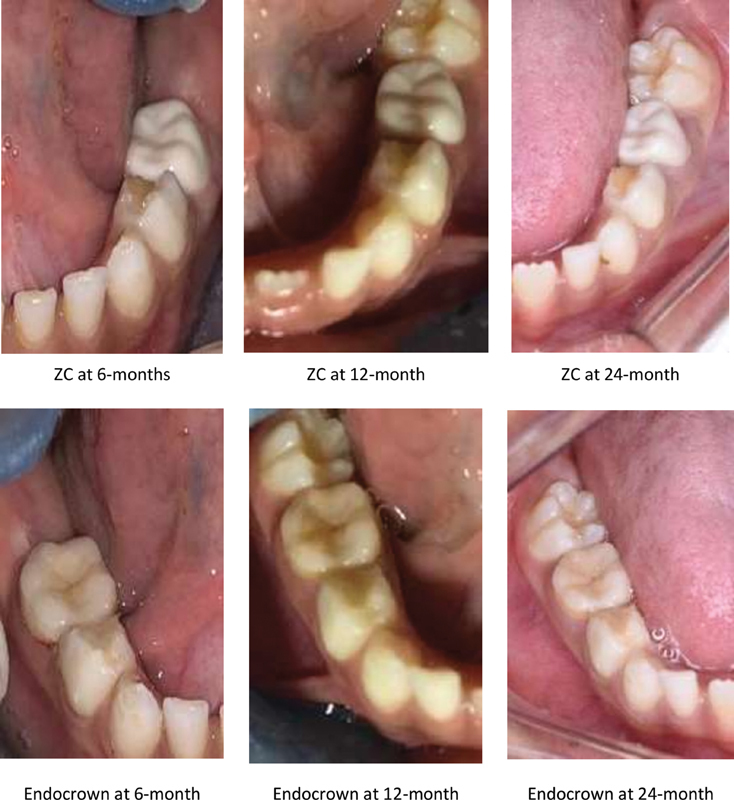
Clinical performance photographs of the endocrown and zirconia crown at 6, 12, and 24 months of the follow-up period for a 5-year-old girl.

The average score of dental plaque accumulation on the ZCs was nearly similar at the follow-ups and less than that recorded with endocrown restorations. PI average scores for ZCs at the T1, T2, and T3 were 0.07 ± 0.20, 0.10 ± 0.37, and 0.11 ± 0.33, respectively. For endocrown restorations, the average PI scores at T1, T2, and T3 were 0.09 ± 0.33, 0.16 ± 0.43, and 0.17 ± 0.48, respectively. The total PI average scores of ZC and endocrown were 0.29 ± 0.93 and 0.40 ± 1.06, respectively ( *p* = 0.59) (mean difference 0.12, 95% CI: −0.31, 0.54). Gingival health at the follow-ups of both restorations was nearly similar with comparable mean scores (mean ± SD: T1 [ZC: 0.07 ± 0.20, and endocrown: 0.07 ± 0.33]; T2 [ZC: 0.20 ± 0.11, and endocrown: 0.04 ± 0 0.20]; T3 [ZC: 0.02 ± 0.14, and endocrown: 0.03 ± 0.18]). The overall mean GI score of ZC was 0.11 ± 0.53 and 0.1 4 ± 0.71 ( *p* = 0.82) (mean difference 0.03, 95% CI: −0.24, 0.30). At 12 and 24 months, mild gingivitis was observed in three teeth restored with the endocrowns (6.8%) and two teeth restored with ZCs (4.5%) ( Table 3 and Fig. 3 ).

**Table 3:** Mean and standard deviation of gingival health in terms of PI and GI of the endocrown and zirconia crown groups at different follow-ups

	Mean (SD)	Mean difference	SE	95% CI	*p* a
PI scores					
At 6 mo					
Endocrown	0.09 (0.33)	0.02	0.06	−0.13; 0.10	0.73
ZC	0.07 (0.20)				
At 12 mo					
Endocrown	0.16 (0.43)	0.06	0.09	−0.23; 0.11	0.48
ZC	0.10 (0.37)				
At 24 mo					
Endocrown	0.17 (0.48)	0.06	0.09	−0.23; 0.11	0.50
ZC	0.11 (0.33)				
GI scores					
At 6 mo					
Endocrown	0.20 (0.33)	0.11	0.06	−0.01; 0.23	0.07
ZC	0.09 (0.21)				
At 12 mo					
Endocrown	0.20 (0.24)	0.09	0.06	−0.02; 0.20	0.11
ZC	0.11 (0.28)				
At 24 mo					
Endocrown	0.14 (0.18)	0.06	0.04	−0.01; 0.13	0.08
ZC	0.08 (0.13)				

Abbreviations: CI, confidence interval; GI, gingival index; PI, plaque index; SD, standard deviation; SE, standard error; ZC, zirconia crown.

a Paired sample *t* -test, level of significance (α) was set to 5% ( *p* < 0.05 for two-tailed test).

The overall parental acceptance of both types of restorations was statistically insignificant ( *p* = 0.07). The parents were more satisfied with the endocrown color over the ZC ( *p* < 0.05) ( Table 4 ).

**Table 4:** Frequency of parent's satisfaction scores of endocrown and zirconia crown groups at 24-month follow-up

Parental satisfaction items	Endocrown					ZC					*p* a
	Very dissatisfied	Dissatisfied	Neutral	Satisfied	Very satisfied	Very dissatisfied	Dissatisfied	Neutral	Satisfied	Very satisfied	
Color	0 (0)	0 (0)	1 (2.3)	2 (4.5)	41 (93.2)	0 (0)	0 (0)	2 (4.5)	6 (13.6)	36 (81.8)	0.05 a
Shape	0 (0)	0 (0)	1 (2.3)	1 (2.3)	42 (95.5)	0 (0)	0 (0)	2 (4.5)	1 (2.3)	41 (93.2)	0.36
Size	0 (0)	0 (0)	1 (2.3)	1 (2.3)	42 (95.5)	0 (0)	0 (0)	2 (4.5)	1 (2.3)	41 (93.2)	0.84
Overall satisfaction	0 (0)	0 (0)	1 (2.3)	2 (4.5)	41 (93.2)	0 (0)	0 (0)	4 (9.1)	3 (6.8)	37 (84.1)	0.07

a Friedman test, level of significance (α) was set to 5% ( *p* < 0.05 for two-tailed test).

## Discussion

Aesthetically restoring pulpotomized primary molars continues to be a challenging aspect in pediatric dentistry. Preserving the tooth structure while ensuring the high quality of aesthetic standards is an imperative need, especially with the exceptional progress in adhesive dentistry. The current trial was performed to compare the clinical performance of glass-ceramic endocrowns as a new restoration of pulpotomized primary molars compared with prefabricated ZCs. Compared with ZCs, ceramic endocrowns need less circumferential tooth reduction, may be more aesthetic, have superior morphological and anatomical properties, and attain retention through macro- and micromechanical means. 6 23

For standardization, the current study accredited the split-mouth technique using the two contralateral mandibular primary molars. Such a design guarantees several merits. For instance, (1) expose both restorations to similar oral circumstances and allow comparable periodontal health with different treatment modalities, 24 and (2) from the ethical point of view, no child was denied the benefits of either type of restorations.

Among glass-ceramic restorations, LS2 ceramic (IPS e.max Press/Ivoclar Vivadent) is considered the material of choice in the current study because of its superior mechanical properties (fracture toughness of 2–3 MPa and flexural strength of 360–440 MPa). Moreover, its content of LS2 provides a bond strength with the cavity walls. For these advantages, this material is considered the gold standard among all glass-ceramic restorations. 25

Clinical performance was assessed using a modified USPHS criterion because of its wide use and it is a well-accepted method for clinical evaluation. 11 26 Gingival and plaque status was assessed using GI and PI indices. These indices have proven to be accurate and reproducible tools in clinical research. 27 Our findings showed very good gingival health among the ZC group at the follow-ups and excellent parental acceptance. This was in agreement with the findings of Walia et al, who reported a 100% clinical success rate of ZCs. 2

Until now, there are no literatures that discussed the LS2 endocrown restoration success rate in primary molar teeth. Up till now, all data are concerned with permanent teeth. However, the high clinical success rate of LS2 endocrowns in permanent dentition has been approved by a former study. 28 This high success rate was in line with the results of the present study.

Concerning the effect on gingival health, most of the restorations in the two groups showed no visible dental plaque accumulation after 24 months and only five cases in each group showed a minimal plaque accumulation interpreting as a good oral hygiene. Furthermore, the gingiva was healthy around both restorations at the follow-ups. Only one case showed signs of mild gingivitis in both restorations. For the control group, these results could be attributed to the remarkable biocompatibility and smooth polished exterior of ZCs that resulted in a lower tendency of plaque build-up and subsequent gingival irritation. Earlier publications on fixed partial dentures in the permanent dentition found similar outcomes of decreased plaque build-up. 29

Regarding gingival condition in the endocrown group, one advantage of these restorations is their supragingival preparation which avoids gingival trauma during tooth preparation. Moreover, LS2 exhibits high levels of biocompatibility due to low plaque retention. The high biocompatibility of LS2 has been proved in a previous *ex vivo* study. 30

Parents were more satisfied with the color of endocrowns than ZCs and this response difference was statistically significant. This might be related to the use of split-mouth design which allowed an instant and direct comparison between the two restorations, and the parental opinions were in favor to the LS2 endocrowns. This finding might be due to the availability of choosing the shade of the endocrown restoration, while ZCs are available in two shades only. Therefore, one would hope your shade selection leads to a greater color satisfaction. Another point is the natural look of the LS2 endocrown with its high aesthetic features, especially their translucency, which is approximately 30% higher than the conventional zirconia. 12 In addition, endocrowns fabricated with the pressed LS2 show superior anatomic contour and surface textures. 31 Regarding parental satisfaction with ZCs, the current study findings were in agreement with the results of Salami et al. 32

One of the positive points of using endocrowns is the conservative circumferential tooth reduction that minimizes the variability of tooth reduction amount among operators. The estimated total cost (i.e., crown price, temporization, and bonding/cementation price) of the two restorations was comparable. The price of NuSmile ZC was approximately $31.4 U.S. dollars, and the price of resin cement for bonding was $7.9 (the total price was $39.3). However, the endocrown laboratory work expenses were $35.7, temporization expense was $3.9, and its bonding price was about $7.9 (the total price was $47.5). However, some disadvantages of glass-ceramic endocrowns in comparison to ZCs were addressed, such as (1) the need for an extra appointment and additional time, (2) exposed cervical margins may be at risk of caries especially in children with high-risk caries, (3) additional procedures such as impression, temporization, and laboratory work, and (4) finally, the cementation procedures of the endocrown restoration are more complicated than those for the ZCs.

### Study Strength and Limitations

This study was a leading trial in the use of endocrowns for restoration of pulpotomized primary molars with an adequate sample size and relatively long follow-up period. Moreover, the study design adopted rigorous measures to ensure high quality of standardization. The trial findings provided an additional aesthetic solution for the parents/children to satisfy the growing need of aesthetic restoration of primary molars. However, there is need for more longitudinal randomized controlled trials with longer follow-up periods.

## Conclusions

Within the limitations of the current study, it can be concluded that (1) clinical evaluation of endocrowns in terms of marginal adaptation, marginal discoloration, restoration integrity, and gingival health was very good and comparable to the results of ZCs, and (2) endocrowns meet the aesthetic demands in the mind of parents, especially their color. However, there was no statistically significant difference between the overall parental satisfaction of both restorations, and LS2 endocrowns hold potential; however, they have some clinical limitations to placement.
