# Supplementary material for: Clinical Evaluation of CAD/CAM Ceramic Endocrown Versus Prefabricated Zirconia Crown in the Restoration of Pulpotomized Primary Molars: A Two-Year Spilt-Mouth Randomized Controlled Trial
Source: Eur J Dent. 2022 Feb 23;16(3):627–36. doi: 10.1055/s-0041-1736417 (PMC9507603; doi:10.1055/s-0041-1736417)
Supplement: Supplementary file 1 — Supplementary Material [file 10-1055-s-0041-1736417-s2171657.pdf]

Appendix A1

Appendix Table A1 Clinical performance of endocrowns and zirconia crowns using U.S. Public Health Service (USPHS)

	Endocrowns			Zirconia crowns		
**1.Marginl adaptation**	**Alpha** No lack of continuity along the margin as observed with the explorer	**Bravo** Evidence of a crevice along the margin, but the explorer cannot penetrate	**Charlie** Evidence of a crevice along the margin, into which an explorer can penetrate. Requires control, repair, or retreatment	**Alpha** No lack of continuity along the margin as observed with the explorer	**Bravo** Evidence of a crevice along the margin, but the explorer cannot penetrate.	**Charlie** Evidence of a crevice along the margin, into which an explorer can penetrate. Requires control, repair, or retreatment
At 6 months						
At 12 months						
At 24 months						
**2. Marginal discoloration**	**Alpha** No discoloration anywhere on the margin	**Bravo** Discoloration present but does not penetrate along the margin toward the pulp	**Charlie** Severe discoloration. The restoration must be removed	**Alpha** No discoloration anywhere on the margin.	**Bravo** Discoloration present but does not penetrate along the margin toward the pulp	**Charlie** Severe discoloration. The restoration must be removed
At 6 months						
At 12 months						
At 24 months						
**3. Integrity of restoration**	**Alpha** Completely intact	**Bravo** Crack apparent on transillumination	**Charlie** Fracture observable	**Alpha** Completely intact	**Bravo** Crack apparent on transillumination	**Charlie** Fracture observable
At 6 months						
At 12 months						
At 24 months						

Appendix Table A2 Plaque index scores, criteria, interpretation, and calculation of endocrowns and ZCs over the follow-up period

**Scores and criteria**								
**Score**	**Criteria**							
0	No plaque							
1	A film of plaque adhering to the free gingival margin and adjacent area of tooth the plaque may be seen *in situ* only after application of disclosing solution or by using a probe on the tooth surface							
2	Moderate accumulation of soft deposits within the gingival pocket, or the tooth and gingival margin which can be seen with the naked eye							
3	Abundance of soft matter within the gingival pocket and/or on the tooth and gingival margin							
	**PI score of endocrowns**				**PI score of zirconia crowns**			
	**0**	**1**	**2**	**3**	**0**	**1**	**2**	**3**
At 6 months								
At 12 months								
At 24 months								
**Interpretation**								
Excellent					0			
Good					0.1–0.9			
Fair					1.0–1.9			
Poor					2.0–3.0			

 © 2022. The Author(s).

Calculation

Plaque index for a tooth calculated by dividing the total scores around each tooth (four scores) by the number of surfaces examined (four surfaces).

Appendix Table A3 Gingival index scores, criteria, interpretation, and calculation of endocrowns and ZCs over the follow-up period

<table>
<tr><th colspan="9">Scores and criteria</th></tr>
<tr><th>Score</th><th colspan="8">Criteria</th></tr>
<tr><td>0</td><td colspan="8">Absence of inflammation/normal gingiva</td></tr>
<tr><td>1</td><td colspan="8">Mild inflammation, slight change in color, slight edema, no bleeding on probing</td></tr>
<tr><td>2</td><td colspan="8">Moderate inflammation, moderate glazing, redness, edema, and hypertrophy. Bleeding on probing</td></tr>
<tr><td>3</td><td colspan="8">Severe inflammation, marked redness, and hypertrophy ulceration. Tendency to spontaneous bleeding</td></tr>
<tr><td rowspan="2"></td><th colspan="4">GI score of endocrowns</th><th colspan="4">GI score of zirconia crowns</th></tr>
<tr><th>0</th><th>1</th><th>2</th><th>3</th><th>0</th><th>1</th><th>2</th><th>3</th></tr>
<tr><td>At 6 months</td><td></td><td></td><td></td><td></td><td></td><td></td><td></td><td></td></tr>
<tr><td>At 12 months</td><td></td><td></td><td></td><td></td><td></td><td></td><td></td><td></td></tr>
<tr><td>At 24 months</td><td></td><td></td><td></td><td></td><td></td><td></td><td></td><td></td></tr>
<tr><th colspan="9">Interpretation</th></tr>
<tr><td colspan="5">Normal gingiva</td><td colspan="4">0</td></tr>
<tr><td colspan="5">Mild gingivitis</td><td colspan="4">0.1–1.0</td></tr>
<tr><td colspan="5">Moderate gingivitis</td><td colspan="4">1.1–2.0</td></tr>
<tr><td colspan="5">Sever gingivitis</td><td colspan="4">2.1–3.0</td></tr>
</table>

 © 2022. The Author(s).

Calculation

Gingival index for a tooth calculated by dividing the total scores around each tooth (four scores) by the number of surfaces examined (four surfaces).

Appendix Table A4 Parental satisfactions at 24-month follow-up period for the endocrown versus ZC

Parental Satisfaction Questionnaire

Dear parent
The questionnaire is aimed to assess your satisfaction toward your child's restoration which to provide better and qualified dental services to our precious kids.

Thank you for your time and help

Date: / /
Parent's data:

- **Name (Optional):——————————————————————————**
- **Age:———————————————————————————————**
- **Gender:—————————————————————————————**

Occupation:———————————————————————————

- **Education level:—————————————————————————**

Child's data

- **Name (Optional):——————————————————————————**
- **Age:———————————————————————————————**
- **Gender:—————————————————————————————-**

On a 5-point-Likert scale, please rate your choice from score "1" equals strongly dissatisfied; "2" equals dissatisfied; " 3"equals neutral satisfied; "4" equals satisfied; to score "5" which equals strongly satisfied.

 © 2022. The Author(s).

Color Matching

There is no noticeable difference of the color of your child crown (endocrown/ZC) from adjacent teeth:

1. Strongly dissatisfied	2. Dissatisfied	3. Neutral	4. Satisfied	5. Strongly satisfied

There is a slight shade mismatch:

1. Strongly dissatisfied	.2. Dissatisfied	3. Neutral	4. Satisfied	5. Strongly satisfied

There is obvious shade mismatch:

1. Strongly dissatisfied	2. Dissatisfied	3. Neutral	4. Satisfied	5. Strongly satisfied

Crown Contour

Crown is cosmetic, natural looking:

1. Strongly dissatisfied	2. Dissatisfied	3. Neutral	4. Satisfied	5. Strongly satisfied

Size/shape is acceptable, not ideal:

1. Strongly dissatisfied	. 2. Dissatisfied	3. Neutral	4. Satisfied	5. Strongly satisfied

Crown not esthetic, detracts from appearance of the mouth:

1. Strongly dissatisfied	2. Dissatisfied	3. Neutral	4. Satisfied	5. Strongly satisfied

Overall satisfaction:

1. Strongly dissatisfied	2. Dissatisfied	3. Neutral	4. Satisfied	5. Strongly satisfied

Thank you for your kind response.

 © 2022. The Author(s).
